# The Relevance of the High Frequency Audiometry in Tinnitus Patients with Normal Hearing in Conventional Pure-Tone Audiometry

**DOI:** 10.1155/2015/302515

**Published:** 2015-10-25

**Authors:** Veronika Vielsmeier, Astrid Lehner, Jürgen Strutz, Thomas Steffens, Peter M. Kreuzer, Martin Schecklmann, Michael Landgrebe, Berthold Langguth, Tobias Kleinjung

**Affiliations:** ^1^Department of Otorhinolaryngology, University of Regensburg, 93053 Regensburg, Germany; ^2^Department of Psychiatry and Psychotherapy, University of Regensburg, 93053 Regensburg, Germany; ^3^Clinic Lech-Mangfall, 83734 Agatharied, Germany; ^4^Department of Otorhinolaryngology, University of Zurich, 8091 Zürich, Switzerland

## Abstract

*Objective*. The majority of tinnitus patients suffer from hearing loss. But a subgroup of tinnitus patients show normal hearing thresholds in the conventional pure-tone audiometry (125 Hz–8 kHz). Here we explored whether the results of the high frequency audiometry (>8 kHz) provide relevant additional information in tinnitus patients with normal conventional audiometry by comparing those with normal and pathological high frequency audiometry with respect to their demographic and clinical characteristics. *Subjects and Methods*. From the database of the Tinnitus Clinic at Regensburg we identified 75 patients with normal hearing thresholds in the conventional pure-tone audiometry. We contrasted these patients with normal and pathological high-frequency audiogram and compared them with respect to gender, age, tinnitus severity, pitch, laterality and duration, comorbid symptoms and triggers for tinnitus onset. *Results*. Patients with pathological high frequency audiometry were significantly older and had higher scores on the tinnitus questionnaires in comparison to patients with normal high frequency audiometry. Furthermore, there was an association of high frequency audiometry with the laterality of tinnitus. *Conclusion*. In tinnitus patients with normal pure-tone audiometry the high frequency audiometry provides useful additional information. The association between tinnitus laterality and asymmetry of the high frequency audiometry suggests a potential causal role for the high frequency hearing loss in tinnitus etiopathogenesis.

## 1. Introduction

Tinnitus is the perception of sound without a corresponding external source. Tinnitus can have many forms and various factors can contribute to its etiology. However, it is well established that hearing loss represents the most important risk factor for tinnitus [1]. The majority of tinnitus patients display increased hearing threshold in the pure-tone audiometry (PTA), particularly in the high frequency range [2–4]. Moreover the frequency spectrum of one individual's tinnitus corresponds to the frequency range of the hearing impairment [5, 6], thus underscoring the relevance of hearing loss as an etiologic factor for tinnitus. However, some tinnitus patients present without any detectable hearing loss in the frequency range of the conventional pure-tone audiometry (125 Hz–8 kHz) [7, 8]. It has been argued that a normal pure-tone audiogram (PTA) does not reliably preclude cochlear damage. Damage of hair cells coding for frequencies between the tested frequencies or above 8 kHz is not detected by the conventional audiometry. Accordingly, tinnitus patients with normal audiograms had more frequently cochlear dead regions [9] and outer hair cell damage and impaired hearing thresholds in the extended high frequency region [10] as compared to control groups.

Furthermore, patients with tinnitus and normal audiograms demonstrated a significantly reduced amplitude of the wave I potential in the auditory brainstem response [7], suggesting damage of hair cells and/or auditory nerve fibers already at normal audiometric thresholds. Taken together these studies support the theory of a “hidden hearing loss” in tinnitus patients. However the question remains whether the high frequency audiometry should be recommended as a standard diagnostic procedure in the routine assessment of tinnitus patients [11]. One possible approach for answering this question is the investigation, how much additional clinical information is provided by the results of the HF-audiogram in tinnitus patients? For this purpose we investigated tinnitus patients with normal conventional PTA from the Tinnitus Research Initiative Database and contrasted the groups with normal and increased hearing thresholds in the HF-audiometry with respect to various clinical and demographic characteristics.

## 2. Material and Methods

Clinical, demographic, and audiometric data were obtained as part of the routine assessment at patient intake at the Interdisciplinary Tinnitus Center of the University of Regensburg, Germany, and collected in the Tinnitus Research Initiative Database [12]. Data were analysed from all patients presenting with chronic subjective tinnitus between 2007 and 2012, for which both conventional and HF PTA were available, who had normal hearing thresholds in the conventional PTA and who had given written informed consent for data recording and analyzing. The database studies are approved by the local institutional review board (ethical committee of the University of Regensburg).

The term “normal PTA” was defined as ≤15 dB HL over all frequencies from 125 Hz to 8 kHz [13]. The Tinnitus Sample Case History Questionnaire (TSCHQ) was used to gather clinical and demographical data of all patients [14]. Tinnitus severity was assessed by the German version of the Tinnitus Questionnaire (TQ) [15], the Tinnitus Handicap Inventory (THI) [16], and several numeric rating scales concerning tinnitus loudness/discomfort/annoyance/ignorability and unpleasantness. In addition, the Beck Depression Inventory (BDI) was used for quantification of depressive symptoms [17]. The audiological assessment included conventional PTA (125 Hz–8 kHz), HF-audiometry (at 10 kHz, 11.2 kHz, 12.5 kHz, 14 kHz, and 16 kHz), and matching of the tinnitus pitch. Audiometry and tinnitus matching were done with a Madsen Itera (GN Otometrics, Germany) audiometer with Sennheiser HDA-200 supra-aural headphones (Sennheiser electronic GmbH & Co. KG, Germany). The hearing threshold for all frequencies was determined by a standard Hughson-Westlake procedure (steps: 10 dB down, 5 dB up; 2 out of 3). The mean hearing level (dB HL) was calculated by averaging all thresholds for both ears measured in PTA from 125 Hz to 8 kHz. The same was done for the mean HF-hearing level (dB HL) for all frequencies from 10 kHz to 16 kHz. For tinnitus matching, the lower and upper bound frequency [Hz] of the tinnitus were assessed and the center frequency was determined as the geometric mean of both values.

Patients were divided into two groups: the first group included patients with normal thresholds in the HF-audiogram (≤15 dB HL over all frequencies) (HF-norm); the second group included patients with HF-hearing loss (HF-HL; hearing thresholds over 15 dB HL in at least one frequency). Those groups were compared with respect to gender, age, hearing threshold (range from 125 to 8 kHz), tinnitus severity (TQ, THI, and rating scales), depressive symptoms (BDI), tinnitus laterality, tinnitus duration, tinnitus pitch, presentation of selected somatic symptoms (headache, vertigo, temporomandibular disorder, neck pain, or other pain syndromes), and different triggers for tinnitus onset (loud blast of sound, whiplash, change in hearing, stress, and head trauma). Independent samples *t*-tests, chi-square tests, and Fisher exact tests were used for group comparisons. In addition, the relation between HF-audiogram asymmetry and tinnitus laterality was examined. For this purpose, the average of the HF-audiometry was calculated separately for the left and right ear. An asymmetry index was defined as the difference between the left and right ear with negative values indicating more pronounced hearing loss in the right ear and positive values indicating more pronounced hearing loss in the left ear. This asymmetry index was used as a dependent variable in an analysis of variance with laterality of tinnitus (measured in three categories: left ear, right ear, and bilateral/inside the head) as independent variable. Post hoc *t*-tests were controlled for multiple comparisons using a Bonferroni correction. All statistical tests were two-tailed. A value of *P* < 0.05 was used to determine statistical significance. Data in the text and tables are given as mean ± standard deviation.

## 3. Results

Data from 75 patients (61.5%; 43 men and 32 women; mean age 37.25 ± 10.25) with chronic tinnitus were analyzed. Thirteen of these patients (9 men and 4 women) had a normal HF-audiogram (see Table 1). The independent samples *t*-test comparing the HF-hearing level between both groups is highly significant, reconfirming the allocation of patients with normal versus pathological high frequency audiogram (see Table 1). The other group comparisons were significant for age, the tinnitus questionnaire, and tinnitus handicap inventory (see Table 1). Patients with pathological high frequency audiogram were significantly older and scored higher on the TQ and the THI in comparison to patients with normal high frequency audiogram. These significant results were confirmed, when the cutoff for a normal versus pathological high frequency audiogram was changed from 15 dB to 20 dB. If the cutoff was increased to 25 dB, the group difference in the TQ and THI did not reach significance level any more. The other results remained unchanged.

**Figure 1:**
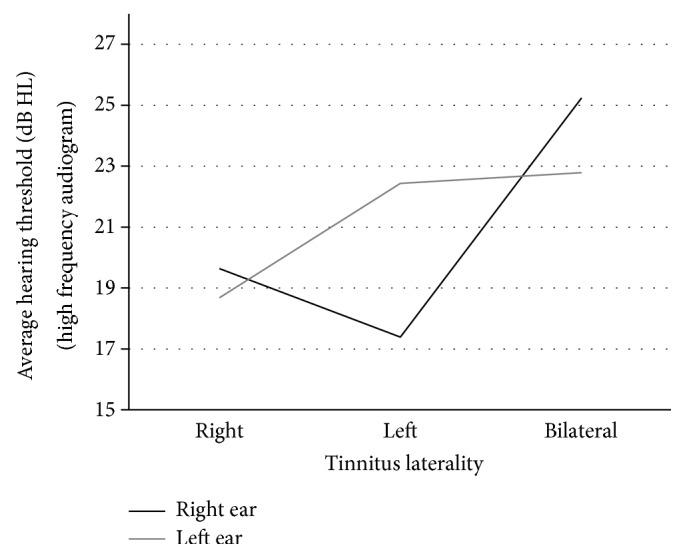
Tinnitus laterality and HF-hearing loss in the right and left ear.

The ANOVA comparing the HF-audiogram asymmetry index for patients with left, right, and bilateral tinnitus was significant (*F*(2,71) = 4.76; *P* = 0.012). Post hoc *t*-tests indicate a significant difference between patients with left and bilateral tinnitus (*P* = 0.012). Patients with left versus right (*P* = 0.086) and with right versus bilateral (*P* > 0.99) tinnitus did not differ significantly. As can be seen in Table 2, patients with left sided tinnitus show positive values in the asymmetry index indicating more high frequency hearing loss in the left ear. Patients with right sided and bilateral tinnitus show negative values, indicating more hearing loss in the right ear. More information about the composition of the asymmetry index can be found in Figure 1, where the average HF-hearing loss for patients with left, right, and bilateral tinnitus is depicted for both ears separately.

## 4. Discussion

The association of chronic tinnitus and hearing loss is well established. Hearing loss is considered to be the most important risk factor for tinnitus [18] and a relationship between the laterality and pitch of tinnitus and the hearing loss could be demonstrated in several studies [3–5].

Since many patients report their tinnitus pitch in the high frequency range, it has been suggested that a comprehensive audiological assessment in tinnitus patients should include HF-audiometry [11]. The purpose of this study was to verify whether the results of the HF-audiometry would provide any additional clinically meaningful information in patients with normal conventional PTA.

First, we found that the majority of our tinnitus patients with normal audiogram had an abnormal HF-audiogram. This fits with earlier findings of increased abnormalities in the HF-audiogram [19] and in the HF otoacoustic emissions [20, 21] in tinnitus patients as compared to controls without tinnitus. Our findings also confirm the notion that the HF-audiometry is more sensitive for detecting hearing damage as compared to the standard audiometry [17, 22, 23]. This fits with our finding that in the HF-hearing loss group a tendency towards worse hearing thresholds in the standard PTA was observed. Given the sensitivity of HF PTA for detecting cochlear damage one may even consider extending the HF PTA to even higher frequencies.

Second, we found a relationship between tinnitus laterality and hearing asymmetry. Patients with left sided tinnitus had also more pronounced HF-hearing impairment on the left side, whereas patients with right sided and bilateral tinnitus had more pronounced HF-hearing impairment on the right side (Table 2). The correspondence between tinnitus laterality and hearing asymmetry for right and left sided tinnitus further confirms the assumption that hearing impairment is involved in tinnitus generation and supports the relevance of HF-audiometry in the diagnosis of tinnitus. The finding of right-accentuated HF-hearing loss in patients with bilateral tinnitus is unexpected and somewhat puzzling. If confirmed by future studies, it suggests that the pathophysiological mechanisms underlying bilateral tinnitus may be distinct from those of unilateral tinnitus. One might have had expected a higher tinnitus pitch in the group with HF-hearing loss. Indeed, in many patients with HF-hearing loss the tinnitus pitch was in the range of the hearing loss. Accordingly, the mean tinnitus pitch was higher in this group. However, due to the high variability of the tinnitus pitch in both groups, this difference did not reach significance level. The demonstration of impaired hearing threshold in the high frequency range in combination with the perception of a high-pitched tinnitus might reflect a very useful element in the counseling of tinnitus patients.

Third, we found that the mean age of the HF-norm group was lower than in the HF-HL group. This is not surprising, since a decline of hearing thresholds with increasing age is well known. The mean age of 24.6 years suggests that a normal HF-audiogram is almost exclusively found in relatively young tinnitus patients.

Fourth, we found higher scores in the TQ and THI questionnaires in the HF-HL group as compared to the HF-norm group. However, this finding should be interpreted with care since this difference did not reach significance any more when the cutoff for normal HF PTA was set at 25 dB HL. Earlier studies have reported higher tinnitus severity in tinnitus patients with more pronounced hearing impairment [24, 25]. In this context it is of interest that also hearing loss in the high frequency range, which should have no direct impact on verbal communication, may result in increased handicap.

One expectation was that other etiologic factors than hearing impairment would be more relevant in people with normal HF-audiometry. However, both groups did differ significantly neither in onset related events like whiplash or stress, nor in comorbidities like neck pain or temporomandibular problems. This may—similarly like the lack of a group difference in tinnitus pitch—be related to a lack of power in the relatively small sample. Moreover, it should be considered that a normal audiogram does not preclude cochlear impairment. Thus, in the group of tinnitus patients with normal standard and HF PTA, dead cochlear regions between the tested frequencies or damage to hair cells or neuronal fibers which are not threshold relevant cannot be excluded.

## 5. Conclusion

To summarize, the results of high frequency audiometry in tinnitus patients with normal conventional PTA are related to tinnitus laterality and tinnitus severity. These findings suggest that the HF-audiometry can be a useful complementary audiological test in a comprehensive diagnostic assessment of tinnitus patients. It should be recommended as a standard procedure in tinnitus patients of younger age including children in the absence of clinical signs of hearing impairment. HF-audiometry might be of therapeutic value within the scope of counseling in explaining the etiopathogenesis of tinnitus to patients with normal conventional PTA but impaired high frequency hearing thresholds.

## Figures and Tables

**Table 1:** Demographic, audiologic, and clinical characteristics of patients with normal versus pathological HF-audiogram.

	*N* (HF-norm/HF-HL^1^)	HF-norm	HF-HL	Group Comparison	
					*P* value
High frequency hearing level (dB HL)	75 (13/62)	2.69 ± 2.49	25.54 ± 12.25	*T*(73) = −13.42	>0.001^*^
Hearing level (dB HL)	75 (13/62)	3.27 ± 1.85	4.40 ± 2.23	*T*(73) = −1.71	0.092
Gender (m/f)	75 (13/62)	9/4	34/28	*χ* ^2^(1,75) = 0.910	0.340
Age	75 (13/62)	24.63 ± 7.10	39.89 ± 8.74	*T*(73) = −5.89	>0.001^*^
BDI	70 (12/58)	7.85 ± 6.00	11.05 ± 9.89	*T*(68) = −1.12	0.267

Tinnitus severity					
TQ	75 (13/62)	23.85 ± 13.95	36.18 ± 17.18	*T*(73) = −2.42	0.018^*^
THI	74 (13/61)	33.69 ± 17.39	48.82 ± 23.61	*T*(72) = −2.66	0.014^*^
Strong/loud	73 (13/60)	4.85 ± 2.30	5.48 ± 2.31	*T*(71) = −0.90	0.370
Uncomfortable	73 (13/60)	6.00 ± 2.24	6.95 ± 2.52	*T*(71) = −1.25	0.214
Annoying	73 (13/60)	4.62 ± 2.40	5.97 ± 2.69	*T*(71) = −1.67	0.099
Unpleasant	73 (13/60)	4.85 ± 2.70	6.03 ± 2.74	*T*(71) = −1.42	0.160
Ignoring	73 (13/60)	5.08 ± 3.07	6.40 ± 2.90	*T*(71) = −1.48	0.144

Tinnitus characteristics					
Laterality (right/left/bilateral, in %)	74 (13/61)	38/31/31	28/31/41		0.691
Pitch	61 (9/52)	7334 ± 2378	7605 ± 4301	*T*(59) = −0.18	0.855
Duration (in months)	73 (13/60)	62.85 ± 95.76	67.68 ± 69.05	*T*(71) = −0.21	0.832

Onset of tinnitus related to no/yes in %					
Sound blast	65 (11/54)	82 /18	93/7		0.266
Whiplash	65 (11/54)	100/0	93/7		>0.999
Change in hearing	65 (11/54)	91/9	94/6		0.533
Stress	65 (11/54)	73/27	43/57		0.099
Head trauma	65 (11)	91/9	98/2		0.312
Others	65 (11)	27/73	48/52		0.320

Comorbidities of tinnitus (no/yes in %)					
Headache	71 (13)	77/23	52/48	*χ* ^2^(1,71) = 2.74	0.098
Vertigo or dizziness	72 (13)	85/15	73/27		0.495
TMD	71 (13)	77/23	62/38		0.358
Neck pain	70 (13)	62/38	46/54	*χ* ^2^(1,70) = 1.07	0.300
Other pain syndromes	71 (13)	92/8	69/31		0.162

Results from independent samples *t*-tests, chi-square tests and Fishers exact tests for group comparisons.

HF-norm: group with normal HF-audiogram; HF-HL: group with HF-hearing loss; m: male; f: female.

^1^Some information was not available for all patients.

^*^ *α* < 0.05.

**Table 2:** Asymmetry in high frequency audiogram for patients with left, right, and bilateral tinnitus.

Tinnitus laterality	*N*	Asymmetry index (left ear−right ear)
Left	23	5.04
Right	22	−0.95
Bilateral	29	−2.45
